# Infection Prevention and Management in Pediatric Short Bowel Syndrome

**DOI:** 10.3389/fped.2022.864397

**Published:** 2022-06-30

**Authors:** Laura Merras-Salmio, Mikko P. Pakarinen

**Affiliations:** ^1^Pediatric Gastroenterology Unit, Children’s Hospital, Helsinki University Hospital, Helsinki, Finland; ^2^Faculty of Medicine, University of Helsinki, Helsinki, Finland; ^3^Department of Pediatric Surgery, Children’s Hospital, Helsinki University Hospital, Helsinki, Finland

**Keywords:** short bowel syndrome, intestinal failure, central venous access, catheter sepsis, parenteral nutrition, intestinal reconstruction

## Abstract

Short bowel syndrome (SBS) is a rare disease with potentially life-threatening consequences. In addition to intestinal failure-associated liver disease, infections and other complications related to central venous catheters (CVCs) cause a significant burden to patients with SBS and may even necessitate an intestinal transplant eventually. The need for long-term central venous access and the intestinal dysfunction associated with SBS drive the need for intestinal failure-specific approach to prevent and treat infections in patients with SBS. In bacterial infections, the line can often be salvaged with proficient antibiotic therapy. Repeated catheter replacements are predisposed to recurrent infections and thrombotic complications, which may limit the long-term survival of patients with SBS. Protocol-based CVC access procedures and daily care including taurolidine and ethanol catheter locks have been shown to reduce infection rates substantially. Compromised intestinal function in SBS predisposes to small bowel bacterial overgrowth, mucosal injury, and increased permeability. These pathophysiological changes are concentrated in a subset of patients with excessive bowel dilatation and frequent bowel-derived infections. In such patients, reconstructive intestinal surgery may be indicated. Probiotics have not been effective in infection prevention in SBS and carry a significant risk of complications. While more studies focusing on the prevention of infections and their complications are needed, protocol-based approach and multidisciplinary teams in the care of patients with SBS have been shown to reduce complications and improve outcomes.

## Introduction

Pediatric short bowel syndrome (SBS) is a rare disease affecting approximately 25–46 out of 100,000 newborns (1, 2). SBS is the major contributor to pediatric intestinal failure, which is broadly defined as a state where the intestine is unable to sustain growth and health without parenteral support (3). Other causes of pediatric intestinal failure include intestinal dysmotility and mucosal diseases. SBS is characterized by the need for prolonged parenteral nutrition (PN) due to significant intestinal loss after resection. In the pediatric population, the major cause of SBS is necrotizing enterocolitis (NEC), followed by intestinal atresias, gastroschisis, and midgut volvulus. In patients with NEC, the prognosis of SBS is often favorable partly because of the remaining bowel growth potential in premature infants. Other important prognostic factors are related to the detailed anatomy of the remaining bowel: longer remaining small intestinal length, the preserved ileum, presence of the ileocecal valve, and remaining colon concur with greater chance of weaning off PN (4).

Liver disease in patients with intestinal failure and on long-term PN manifests typically as cholestasis and as inflammation and fibrosis in young children, whereas steatosis can be observed in any phase of the disease and all age groups (5). The liver of newborns is vulnerable to pathophysiological insults inflicted by infections, disrupted gut-liver axis, and unphysiological delivery of parenteral nutrients, predisposing to development of intestinal failure-associated liver disease (IFALD) (6). Parenteral plant-based lipids, especially soy oil derivatives, contribute to cholestatic liver disease, while fish oil seems to have a protective role (6).

This review was undertaken to provide an update on hospital-based medical and surgical management of patients with pediatric SBS, aiming at an overview of current knowledge of the topic rather than a comprehensive systematic review.

## Therapy and Outcomes of Pediatric Short Bowel Syndrome

The remaining intestine gradually adapts to significant bowel resection. The intestinal adaptation involves hormone-mediated changes in gut microarchitecture, anatomy, motility, and absorptive function. This adaptive process may be modified by medical and surgical approaches. Glucagon-like-peptide -2 (GLP-2) analogs, which have emerged in recent years, are shown to reduce the need of PN in patients with SBS. GLP-2 has a trophic effect on the enterocyte promoting villus length and epithelial barrier function while beneficially affecting gut motility and absorptive functions (7). At present, only teduglutide has been licensed for use in children (8). In addition to routine surgical care of anastomotic strictures or ostomy complications, autologous reconstructive gastrointestinal surgery may be helpful in correctly selected patients in whom propulsive intestinal motility and absorption are hampered by excessive bowel dilation. In the dilated bowel, intestinal lengthening/tapering procedures including serial transverse enteroplasty or the Bianchi procedure may expediate weaning off PN (9).

A mainstay of pediatric SBS treatment is to provide PN and fluid therapy to assure normal growth and development and nutritional status. Multidisciplinary intestinal rehabilitation teams in expert centers have a significant advantage in patient care in terms of optimizing PN, promoting enteral nutrition, and reducing complications such as infections and liver diseases while providing correctly timed listing for intestinal transplant when indicated (10). Even after weaning off PN, children with SBS are at risk for further complications and malnutrition necessitating long-term expert follow-up (11, 12). Important complications of pediatric SBS include infections, associated with both central venous catheter (CVC) and the diseased gut. Different aspects of infection prevention and management in pediatric patients with SBS are depicted in Table 1 and discussed further below.

**TABLE 1:** Infection prevention strategies for pediatric patients with short bowel syndrome (SBS).

Intervention	Proposed mechanism of action	Associated negative consequences	Evidence
Catheter lock solutions (taurolidine, ethanol)	Prevention of microbial biofilm formation inside the catheter	Some concern over systematic side effects of ethanol, possible catheter damage associated with ethanol	Strong evidence in favor of systematic use. Promoted in professional guidelines of ESPGHAN and NASPGHAN.
Probiotics	Temporal alteration of gut microbiota, changes in microbial genome and metabolome. Effects on intestinal epithelial and immunological function.	Reported systemic infections, CVC contamination, D-lactic acidosis	Lack of evidence supporting systematic use. Potential hazards significant.
Intestinal hormone therapy	Glucagon-like-peptide 2 -agonist teduglutide has trophic effects on gut mucosa and absorptive function, resulting in enhanced barrier function and reduced small bowel bacterial overgrowth.	Adverse effects associated with teduglutide include abdominal discomfort, nausea, stoma problems and fluid retention.	Strong evidence to support use in PN dependent SBS patients.
Autologous intestinal reconstruction surgery (AIRS), including STEP and other bowel lengthening procedures	Effective tapering of dilated bowel reduces infection rates and may promote motility thus lessening SBBO. Bowel lengthening procedures may reduce PN dependency.	Risks inherent to laparotomy and major bowel surgery, need for re-surgery in case of re-dilation. Correct patient selection remains problematic	Evidence from case series and historical controls support use of AIRS in selected cases. Long term follow-up studies are lacking.
Enteral cyclic antibiotics	Reduce small bowel bacterial overgrowth. Certain antibiotics (e.g., erythromycin, amoxicillin) may have propulsive effect.	May contribute to antibiotic resistance and enrichment of intestinal pathogenic bacteria.	Poor research evidence. Clinical short-term benefit often good. Long term problems described.
Protocol based CVC care, addressing insertion, tunnel skin care and infection prevention and management	Systematic use of all above prevention strategies ensures that all SBS patients receive highest quality of care, preferably in or overseen by an experienced intestinal rehabilitation center	Not reported	Evidence from case series with historical cohorts support the use of guidelines and protocols to guide clinical decision making. Several studies have confirmed the superiority of multidisciplinary care teams for SBS patients, as well as CVC care.

*CVC, central venous catheter; SBS, short bowel syndrome; PN, parenteral nutrition; STEP, serial transverse enteroplasty.*

## Central Line-Associated Infections in Pediatric Patients With Short Bowel Syndrome

The CVC access is pivotal in managing children with SBS dependent on PN. Central access allows for more concentrated PN and/or larger volumes of fluids while simultaneously precluding repeated cannulations. Mostly used CV access involves a tunneled CV line inserted via the external or the internal jugular vein (or other upper central veins, e.g., the Subclavian vein) to the cavo-atrial junction (13). Femoral access is more controversial and, in clinical practice, more problematic (14). Peripherally inserted central lines may also be used for a short term, but they carry higher infection risks. On the rare occasion when venous occlusions prevent any other approach, more challenging approaches may need to be considered. These would include a transhepatic access and direct insertion of the catheter into to the right atrium (transthoracic intracardiac line). PORT-A-CATH-type subcutaneous access devices are not used routinely in most IF centers, as repeated skin punctures to reach the port produce pain and anxiety and are associated with increased risk of infections and extravasation in case of needle misplacement (13).

For the pediatric patients with IF, every catheter replacement raises the risk for central vein occlusion and eventual loss of venous access. Catheter infection rates are highest for a couple of months after each CVC insertion (15, 16). Occlusion of the tunneled CVC also occurs in up to 12% of patients (17). In patients with IF, infection prevention not only reduces infection-related morbidity and mortality but also has long-term implications for the patients as venous access is crucial for survival in severe SBS.

Pediatric patients with SBS are seldom immunocompromised; thus, catheter-related blood stream infections (CRBSIs) are mostly caused by commensal skin or intestinal bacteria. In newborns or patients with concomitant immunodeficiencies, there may be a more varied etiology. Local antibiotic resistance situation and antibiotic availability affect the choice of empiric treatment, which should be directed against Gram-positive cocci and enterobacteria (13). Blood cultures are always needed to guide further therapy.

After insertion of the CV catheter, a biofilm of microbes quickly develops on the surface of the catheter. This biofilm is the origin of most CRSBI pathogens, which are typically Gram-positive staphylococci or intestinal-origin enterobacteria, or less frequently fungi (18). The CV catheter lock prophylaxis prevents the growth of this biofilm and thus effectively reduces CRBSIs. Antibiotic-based locks have not been effective in preventing catheter-related infections, and they also carry the risks of antibiotic resistance and systemic effects. However, antimicrobial lock solutions such as taurolidine and ethanol have been used in pediatric patients with CVC with significant success (18). A recent open label study on taurolidine lock therapy included 33 pediatric CV line-dependent patients with at least one previously confirmed line infection. After a median duration of 140 days, taurolidine lock therapy showed 80% reduction in CRBSIs compared to pre-lock infections rates (19). Also, in a randomized controlled study on 41 adult patients with IF with significant history of previous CRBSIs, combined taurolidine-heparin locks were 100% efficacious in median 337 days in preventing CRBSIs when compared to heparin locks alone (20). Furthermore, taurolidine therapy seems safe and its protective effect durable on patients with long term CVCs (21). Ethanol locks also have high efficacy to prevent CRBSIs, as has been shown in both adult and pediatric HPN patients (22, 23). However, some concern has been raised over a possibly increased risk of thrombosis, catheter damage, and systemic toxicity associated with ethanol locks (24). The European Society for Pediatric Gastroenterology, Hepatology and Nutrition (ESPGHAN) stated a strong recommendation for taurolidine lock use in its 2018 PN Guidelines (25). The NASPGHAN IF interest group also recently recommended prophylactic lock therapy with non-antibiotic locks in all children with high risk of CRSBIs (13).

The tunneled CVC has the disadvantage of the skin tunnel to the venous insertion site, which is vulnerable to chronic skin infections caused by *Staphylococcus aureus* and, more infrequently, by other bacteria. Therefore, tunnel exit site care needs to be addressed. Topical chlorhexidine containing prophylactic wound care products are recommended by the ESPGHAN for children over 2 months of age, as it is shown to reduce skin infections (25). Still, recurrent aureus infections may be problematic. In pediatric dialysis patients with chronic aureus infections, intranasal topical mupirocin has been effective in reducing nasal aureus carriage and more severe aureus-related infectious complications (26). This approach might also be beneficial in children with IF. Recurrent or severe tunnel infection is an indication for CV line replacement (13). Multidisciplinary IF care teams should also include dedicated venous access specialists who may be surgeons, interventional radiologists, or anesthesiologists, and experienced nursing specialists (25, 27).

## Intestinal Origins of Infections in Short Bowel Syndrome

In SBS, the remaining bowel mucosa is often compromised by inflammatory changes (including villous atrophy) and impaired barrier function (28). Symptoms of small bowel bacterial overgrowth (SBBO) occur frequently, especially in patients with excessively dilated bowel in combination with signs of mucosal damage. Unfortunately, clinically feasible testing to diagnose SBBO in young children is lacking, and the diagnosis of SBBO is mostly based on clinical suspicion (29). SBBO significantly hampers weaning off PN and may promote IFALD, and increases the likelihood of blood stream infections by seven-fold (30). The excessively dilated small bowel diameter alone increases infection rates by two-fold, while surgical tapering interventions reduce the infection rates significantly (31). SBBO has been classically treated with antibiotics, but in clinical practice patients seem to become dependent on cyclic antibiotic regimens and may develop wide spectrum-resistant bacterial and fungal infections. This observation was recently confirmed in a study addressing gut microbes in pediatric SBS proving antibiotic-driven gut dysbiosis with ensuing enrichment of pathogenic intestinal bacteria (32). Therefore, in recurrent clinically suspected SBBO, evaluation of the degree of bowel dilatation and suitability for autologous reconstructive surgery is prudent, although optimal selection of patients and the surgical procedure remains challenging. Gut dysmotility is a relatively frequent complication of severe SBS. In addition to excessive bowel dilatation, it may be primarily caused by underlying pathologies such as gastroschisis or ischemic injury in patients with NEC (33). Use of antibiotics such as erythromycin and amoxicillin to alleviate dysmotility is not uncommon, as they are known to have a propulsive efficacy (34). These antibiotics may combine two mechanisms of action, direct propulsive effect and microbiome remodeling, to treat SBBO.

As to the mucosal function in SBS, infant patients with SBS (*n* = 7, median age 7 months) who had intestinal permeability evaluated by lactose-mannitol test all showed compromised permeability (30). In a series of 33 pediatric patients with SBS (median age 3 years), duodenal mucosal biopsies showed histologic and molecular signs of both inflammation and increased permeability. Intestinal epithelial barrier function was analyzed by depicting the mucosal mRNA expression of barrier function-regulating genes (35). In pediatric patients with SBS, liver diseases are associated with infections. Higher cases of septic infections have been linked convincingly to histopathological IFALD, while most infections were caused by enteric bacteria in patients with excessively dilated bowel (31). In patients with SBS, the disturbed gut mucosal barrier likely contributes to liver damage by translocation of lipopolysaccharides and other bacterial antigens (6). Development of liver steatosis is associated with aberrant intestinal microbiota characterized by abundance of lipopolysaccharide-producing proteobacteria in PN-dependent children, although the full scale of gut-liver crosstalk in SBS is not fully understood (31, 36).

Probiotics are proposed to suppress pathogenic intestinal bacteria while promoting beneficial commensal microbes. Such changes could be beneficial to patients with SBS in terms of reducing intestinal complications, infection rates, and even liver damage. In a recent study, the probiotic strains of lactobacilli given to nine pediatric patients with SBS for 2 months had no sustainable effects on patient wellbeing or intestinal microbiota (37). This lack of clinical effect is in line with previous studies (38). The impaired mucosal barrier function in SBS means that any probiotic strain has a greater chance of translocation into the bloodstream. Systemic infections and episodes of D-lactic acidosis associated with probiotic use in SBS have been reported (39–41). Without robust evidence of their benefits, the risk of harm is such that use of probiotics in patients SBS should be cautioned. Most probiotic strains show wide-spectrum antibiotic resistance (42). Furthermore, different probiotic strains are shown to have different effects on the gut microbiota and human health (43).

## Discussion

Pediatric patients with IF have a much better prognosis today considering the declining occurrence of IFALD and more refined HPN programs. However, infections originating both in the diseased intestine and central venous catheters remain critical. While CV access-related infections have been shown to abate when utilizing strict handling protocols and antimicrobial locks, the compromised gut mucosal function is still harder to address.

In all treatment strategies concerning pediatric patients with IF’ CRBS or tunnel infections, catheter replacement should be the last option. Most catheters may indeed be salvaged by prompt and lengthy antibiotic therapy tailored according to microbial sensitivity. Exceptions are infections caused by fungi and severe tunnel infections warranting catheter removal. The modern care of pediatric patients with IF should include antimicrobial lock therapy, locally adjusted protocols depicting CV access procedures, CV tunnel insertion care, and infection management (13, 25). The care of CV catheters in children with SBS should aim at long-term patency of the venous access from the beginning.

As to the intestinal function in relation to infections, there are more puzzles to solve. Even in general, SBBO diagnosis and treatment continue to be problematic. In the recent American College of Gastroenterology SBBO Guideline, all of the six recommendations were labeled as low of evidence (29). Nevertheless, clinical suspicion of SBBO in patients with SBS is frequent (28). Small bowel dilatation has been shown to be associated with both SBBO and risk of sepsis (31). Furthermore, progressive IFALD also seems to be related to septic infections, with some evidence pointing toward the gut-liver-axis as mediator (36). Therefore, infection prevention protocols should also include investigating the intestinal status as well as CV line care in pediatric patients with SBS. More research is needed to address the benefits and harms of using probiotics in patients with SBS, and considering significant adverse event reports, their use is cautioned (38).

A protocol-based approach and multidisciplinary teams in the care of patients with SBS reduce complications and improve outcomes. More studies focusing on the prevention of infections (of both CVC and intestinal origin) and their complications in pediatric patients with SBS are urgently needed.

## Author Contributions

LM-S wrote the first draft of the manuscript. MP critically commented the final version to major extent. Both authors read and approved the final draft of the manuscript.

## Conflict of Interest

The authors declare that the research was conducted in the absence of any commercial or financial relationships that could be construed as a potential conflict of interest.

## Publisher’s Note

All claims expressed in this article are solely those of the authors and do not necessarily represent those of their affiliated organizations, or those of the publisher, the editors and the reviewers. Any product that may be evaluated in this article, or claim that may be made by its manufacturer, is not guaranteed or endorsed by the publisher.
